# Clearance and Utilization of Dicarbonyl-Modified LDL in Monkeys and Humans

**DOI:** 10.3390/ijms241310471

**Published:** 2023-06-21

**Authors:** Vadim Z. Lankin, Galina G. Konovalova, Sergey P. Domogatsky, Alla K. Tikhaze, Igor N. Klots, Marat V. Ezhov

**Affiliations:** 1 Department for Free Radical Biochemistry, E.I. Chazov’ National Medical Research Center of Cardiology, Russian Ministry of Health, Moscow 121552, Russia; gavakon5050@mail.ru (G.G.K.); spdomo@yandex.ru (S.P.D.); allatikhaze@yandex.ru (A.K.T.); 2 Research Institute of Medical Primatology, National Research Center “Kurchatov’ Institute”, Sochi 354376, Russia; igor-imp@mail.ru; 3 Laboratory of Lipid Disorders, E.I. Chazov’ National Medical Research Center of Cardiology, Russian Ministry of Health, Moscow 121552, Russia; marat_ezhov@mail.ru

**Keywords:** oxidative stress, dicarbonyl-modified LDL, malondialdehyde (MDA), glyoxal, methylglyoxal, atherosclerosis

## Abstract

The kinetics of elimination of various dicarbonyl-modified low-density lipoproteins from the bloodstream of *Macaca mulatta* monkeys were investigated. The low-density lipoproteins (LDL) in the monkey blood plasma were isolated by density gradient ultracentrifugation and labeled in vitro with the fluorescent dye FITC; thereupon, they were modified with different natural low molecular-weight dicarbonyls: malondialdehyde (MDA), glyoxal, or methylglyoxal. The control native FITC-labeled LDL and dicarbonyl-modified FITC-labeled LDL were injected into the monkey’s ulnar vein; thereafter, blood samples were taken at fixed time intervals during 24 h. The plasma level of FITC-labeled LDL was determined with spectrofluorimetry. The study established that glyoxal- and monkeysglyoxal-labeled LDL circulated in monkey virtually at the same time as native (non-modified) LDL. In contrast, MDA-modified LDL disappeared from the blood extremely rapidly. Administration of the PCSK9 inhibitor involocumab (which increases LDL utilization) to patients with coronary heart disease (CHD) was found to significantly reduce levels of MDA-modified LDL.

## 1. Introduction

It is firmly established that oxidative stress is an important etiological and pathogenic factor in atherosclerosis and diabetes mellitus [1,2,3,4,5,6]. The development of oxidative stress is paralleled with the augmented production of reactive oxygen species (ROS) and the down-regulation of their utilization by the antioxidant enzyme systems in cells and tissues [1,4,5,6]. The oxidative stress is manifested by the activation of free radical oxidation (FRO) processes in lipid-protein supramolecular complexes (biomembranes and lipoproteins), resulting in the accumulation of lipohydroperoxides, which are the primary products of polyene lipid FRO, whose downstream oxidative destruction culminates with the formation of malondialdehyde (MDA) [5,7]. Hyperlipidemia, an indispensible feature of atherogenesis, up-regulates FRO, resulting in the accumulation of primary and secondary FRO products in blood plasma [2,5,6]. The secondary products of polyene lipids FRO, exemplified by low molecular weight dicarbonyls such as MDA, can efficiently react with free ε-amino groups of lysine in B-100 apolipoprotein in low-density lipoproteins (LDL), which are the blood plasma lipids-transporting nanoparticles, thereby modifying their structure [2,5,6]. Earlier studies by us and other researchers [8,9] have shown that chemically modified LDL, such as MDA-modified LDL, is captured by cultured macrophages together with the scavenger receptors, characterized by a far greater efficiency than that of non-oxidized LDL [8,9]. Importantly, the rate of uptake of truly oxidized LDL [i.e., LDL enriched with acylhydroperoxide (LOOH-LDL) in phospholipids of the LDL outer layer catalyzed by reticulocyte C-15 lipoxygenase] by macrophages does not significantly differ from that of native (non-oxidized) LDL, whereas uptake of MDA-modified LDL is going on at an extremely high rate [8]. In diabetic hyperglycemia, the processes of oxidative glucose transformations are up-regulated, resulting in the accumulation of low molecular weight dicarbonyls structurally similar to MDA, such as an MDA homolog, glyoxal, formed during auto-oxidation of glucose [10], and the MDA isomer, methylglyoxal, which is produced during enzymatic oxidation of triozophosphates [5,11] and after free-radical co-oxidation of lipids and 6-carbohydrates [8,12]. It should also be noted that methylglyoxal can be generated non-enzymatically when phosphorus derivatives are attacked by peroxyl radicals [13]. It has been demonstrated that glyoxal-modified LDL produced during the oxidative catabolism of hydrocarbons is captured by cultured human macrophages with greater efficiency than MDA-modified LDL [11]. These observations have been powerful arguments for stating that the molecular mechanisms of vascular wall lesions in diabetes mellitus are virtually the same as in atherosclerosis, although the latter develops more intensively due to a greater “atherogenicity” of glyoxal- or methylglyoxal-modified LDL [2,4,12]. Remembering that dicarbonyl-modified LDL can be utilized with the help of special receptors, including the scavenger receptors of macrophages [14] and LOX-1 of endotheliocytes [15,16,17,18], it can be *a priori* assumed that such LDL should be eliminated from circulating blood at a pronouncedly higher rate than the non-oxidized LDL. Despite the available literature reporting a few data points on the clearance of native LDL in humans [19,20], we could not find any data on the elimination rate of dicarbonyl-modified LDL from human circulation. There have been limited studies on the clearance of non-oxidized LDL in primates [21,22], and there are no data on the elimination of dicarbonyl-modified LDL from the circulation of these animals. Previously, we demonstrated that the elimination of glyoxal- or methylglyoxal-modified LDL from circulation in humans and rabbits proceeded at the same rate as that of the native (non-oxidized) LDL [23]. At the same time, human or rabbit MDA-modified LDL were eliminated from rabbit circulation at an extremely high rate [23]. Evidently, the LDL transport of the lipids in herbivorous animals such as rabbits can essentially differ from the LDL-dependent lipid transport in omnivorous ones, humans included. From the above reasoning, the aim of this study was the examination of the clearance of the labeled and modified LDL from circulation in *Macaca mulatta* monkeys. These LDL were isolated from the blood plasma of the same monkey species and labeled with FITC, followed by modification with MDA, glyoxal, or methylglyoxal. The importance of this study is determined by the hypothetical key role of dicarbonyl-modified LDL in the molecular mechanisms of vascular wall lesions in atherogenesis and the development of endothelium failure [5,6]. In addition, the clearance of MDA-modified human LDL was investigated as part of a clinical trial on cholesterol-lowering therapy in coronary heart disease (CHD) patients with the PCSK9 inhibitor evolocumab.

## 2. Results

### 2.1. Clearance of Dicarbonyl-Modified LDL in Macaca Mulatta Monkeys

Table 1 shows the data on the elimination of FITC-labeled dicarbonyl-modified LDL from the bloodstream of *Macaca mulatta* monkeys at the early stages after intravenous injection. Actually, 6 min after injection, the blood levels of FITC-labeled native, glyoxal-, and methylglyoxal-modified LDL did not significantly differ from each other. At the same time, the content of MDA-modified LDL was smaller almost by an order of magnitude in comparison with the native (non-modified) LDL (Table 1). On postinjection minute 90, the contents of native as well as glyoxal- and methylglyoxal-modified LDL did not significantly differ in comparison with corresponding levels measured 6 min after injection (Table 1). Importantly, the level of MDA-modified LDL decreased by 18% in 6 min after intravenous injection of FITC-labeled MDA-modified LDL (Table 1).

**Figure 1:**
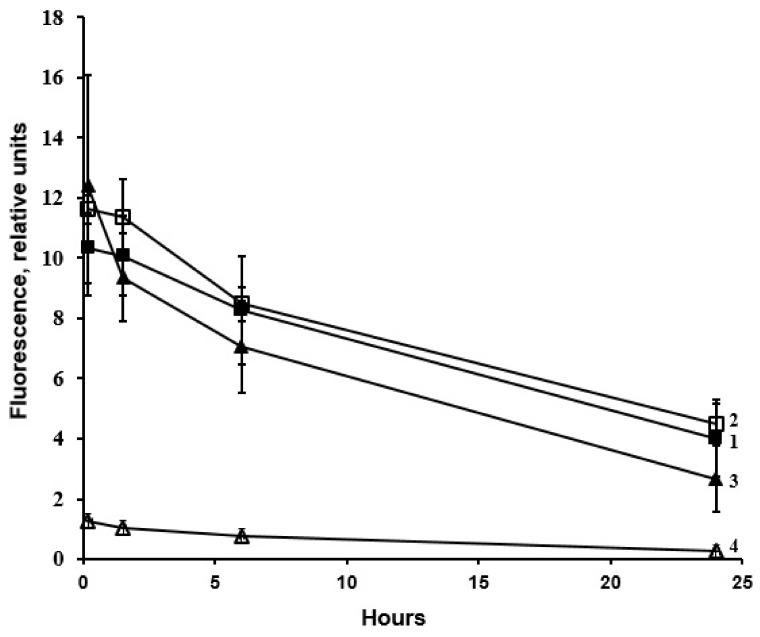
Clearance of FITC-labeled dicarbonyl-modified LDL in *Macaca mulatta* monkeys: 1—native LDL; 2—glyoxal-modified LDL; 3—methylglyoxal-modified LDL; 4—MDA-modified LDL (*differences between bars 1–3 are not statistically significant*; n = 3; *p* > 0.05).

The content of native, glyoxal-, and methylglyoxal-modified LDL in the circulation of *Macaca mulatta* monkeys decreased progressively during the experiments. Actually, a day after injection (at the end of the observation period), the contents were 38.9, 38.7, and 21.4%, correspondingly, relative to initial levels (Table 1; Figure 1, curves 1–3). There were no significant differences in clearance of native as well as glyoxal- and methylglyoxal-modified LDL (Figure 1, curves 1–3). Intravenously injected MDA-modified LDL disappeared from the monkeys’ bloodstream almost completely one day after injection (Figure 1, curve 4).

### 2.2. Effect of the PCSK9 Inhibitor Evolocumab on the Level of MDA-Modified LDL in the Bloodstream of CHD Patients

**Figure 2:**
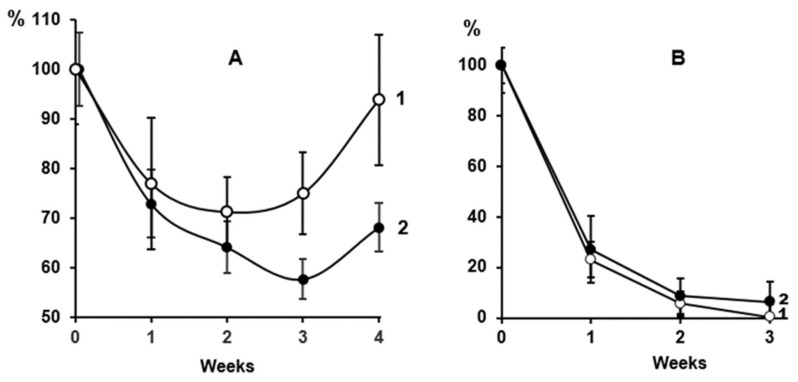
Changes in the content of cholesterol and MDA-modified LDL in the blood of CHD patients during cholesterol-lowering therapy with a PCSK9 inhibitor (evolocumab); n = 9. (**A**): 1—cholesterol LDL content; 2—MDA-modified LDL content (baseline values of each parameter were taken as 100%); (**B**): Dynamics of decreasing during 3 weeks (decrease % per 1 week): 1—LDL cholesterol; 2—MDA-modified LDL.

In the clinical part of the study, we used a conventional method to determine LDL cholesterol levels [24] after a single injection of the PCSK9 inhibitor involocumab, which increases LDL utilization in the liver. In addition, the level of MDA-modified LDL was determined with Mercodia oxidized LDL ELISA kits as described above. Experiments showed that evolocumab significantly and pronouncedly decreased the levels of total cholesterol and LDL cholesterol (Figure 2A, curve 1) in the blood of CHD patients during an observation period of 4 weeks [24]. At the same time, evolocumab significantly and pronouncedly decreased the content of MDA-modified LDL (Figure 2A, curve 2).

The comparative data on the effect of evolocumab on the content of LDL cholesterol and that of MDA-modified LDL (detected with the Mercodia kit) in the blood of CHD patients are shown in Figure 2A, (curves 1 and 2, correspondingly). Figure 2A shows that the levels of LDL cholesterol and MDA-modified LDL decreased within 3 weeks after the administration of evolocumab. Based on these data, we calculated the dynamics of decreasing the studied parameters over 3 weeks (Figure 2B). We can see a striking similarity in the dynamics of decreasing (decrease in % for 1 week) for 3 weeks in the content of total LDL and MDA-modified LDL in the blood of CHD patients after the administration of evolocumab (Figure 2B).

## 3. Discussion

It is an accepted view that the daily LDL turnover in humans is about 25–30% [19,20]. In contrast, our data attest that about 50% of native LDL is eliminated from *Macaca mulatta* circulation during a day. In other words, the clearance of native LDL in *Macaca mulatta*.

In monkeys, it is almost 2-fold greater than in humans. Examination of clearance of native and dicarbonyl-modified LDL in *Macaca mulatta* monkeys showed that FITC-labeled glyoxal- and methylglyoxal-modified LDL were eliminated from blood flow at the same rate as the native FITC-labeled LDL, while the content of FITC-labeled MDA-modified LDL dropped anomalously rapidly as early as the first minutes after intravenous injection, being completely eliminated from the bloodstream on the day after injection (Table 1, Figure 1).

Overall, the data obtained on clearance of dicarbonyl-modified LDL in *Macaca mulatta* monkeys (Table 1, Figure 1) corroborate the early results on clearance of rabbit and human FITC-labeled dicarbonyl-modified LDL in rabbits after a single injection [23]. Both that [23] and the present study (Figure 1) showed that the rate of elimination of FITC-labeled glyoxal- and methylglyoxal-modified LDL from the blood flow of the animals virtually did not differ from the elimination rate of native (non-modified) LDL. In contrast, the FITC-labeled MDA-modified LDL were eliminated from rabbit circulation extremely rapidly, as early as the first minutes postinjection [23]. It is noteworthy that the kinetic curves of the rate of elimination of human and rabbit FITC-labeled dicarbonyl-modified LDL from rabbit bloodstreams were similar [23]. Such a spectacular coincidence of the data obtained on herbivorous (rabbits) and omnivorous (monkeys) animals (Table 1, Figure 1) suggests that elimination of dicarbonyl-modified LDL from blood flow is controlled in mammals by the same mechanism. It should be noted that the modification of LDL under carbonyl stress is probably more atherogenic than that under oxidative stress because the cultured macrophages more actively capture the LDL that has been modified with dicarbonyls formed during oxidative transformations of glucose [11]. At this point, glyoxal- and methylglyoxal-modified LDL circulate in the blood flow for a far longer time than MDA-modified LDL (Figure 1) [23].

Our studies showed that Mercodia oxidized LDL ELISA kits reliably detect MDA-modified LDL in blood plasma with high significance because they contain mAb-4E6 monoclonal antibodies against MDA-modified LDL [25]. Therefore, we used these test kits to examine the utilization of LDL in CHD patients after injection of a Proprotein Convertase Subtilisin/Kexin type 9 (PCSK9) inhibitor known to be implicated in cholesterol homeostasis (Figure 2). It is established that after binding to the LDL-receptor complex, PCSK9 induces its degradation, thus reducing clearance of LDL from the blood [26,27]. Numerous clinical studies showed that therapy with PSCK9 inhibitors pronouncedly decreased the total and LDL cholesterols in the blood of patients with atherosclerosis by up-regulating LDL utilization in the liver [24,28,29].

Figure 2 shows the effect of evolocumab (a PCSK9 inhibitor) on changes in the clearance of LDL and MDA-modified LDL in CHD patients. It progressively decreased the blood levels of LDL cholesterol (Figure 2A, curve 1) and MDA-modified LDL (Figure 2A, curve *2*) assayed with Mercodia oxidized LDL ELISA kits. Evidently, evolocumab simultaneously decreased clearance of LDL and MDA-modified LDL (Figure 2B). Similar results were also obtained in our previous study on rabbits [23]. Based on these data, one can conclude that the anomalously rapid elimination of MDA-modified LDL from mammal circulation results from an extremely high rate of catabolism of these LDL in the liver [23]. The data obtained on primates (Figure 1 and Figure 2) corroborate our previous hypothesis [23]. Thus, one can assume that because of rapid catabolism, MDA-modified LDL plays a minor role in the induction of atherosclerotic vascular wall lesions compared to glyoxal- and methylglyoxal-modified LDL, which circulate in mammal blood flow for a rather long time (Figure 1) [23]. At the same time, the differences in the elimination rate of different dicarbonyl-modified LDL from the mammalian bloodstream can be explained by the different efficiency of binding of these LDL to the vascular endothelium with the participation of the scavenger receptor LOX-1 endotheliocytes [4,5]. The harvested data support our previous hypothesis on a common molecular atherogenic mechanism underlying the development of atherosclerosis and diabetes mellitus, which explains the rapid progression of atherosclerotic lesions in the vascular wall of diabetic patients [1,5]. Presumably, the differences in clearance of MDA-modified LDL, on the one hand, and that of glyoxal- and methylglyoxal-modified LDL, on the other hand, can be explained by the various rates of interaction of these LDL with endothelial scavenger receptor LOX-1, which actively binds with oxidatively modified LDL [15,16,17,18] as evidenced by our previous data [5,6]. Here, the data reliably demonstrated the essential differences in clearance of dicarbonyl-modified LDL formed during lipidemia in the process of atherogenesis (MDA-modified LDL) and those generated during diabetic hyperglycemia (glyoxal- and methylglyoxal-modified LDL), indicating the specific involvement of various dicarbonyl-modified LDL in the molecular mechanisms of atherosclerotic damage to the vascular wall and endothelial dysfunction. It should be noted that oxidatively modified LDL is subject to aggregation, and macrophages cannot fully utilize oxidized LDL, which leads to the formation of foam cells [30]. According to our earlier data [11], the aggregation of LDL after its modification with dicarbonyls also increases.

Evidently, the present data can be used as the basis for novel approaches for the development of medicamentous therapy of atherosclerosis and diabetes mellitus, which should employ not only antioxidants to diminish LDL oxidability [5], but also pharmacotherapy, which includes scavengers of various natural dicarbonyls such as biguanides, imidazole-containing peptides, etc. to block the action of the low molecular weight products of free radical oxidation of lipids and glucose [5].

## 4. Materials and Methods

### 4.1. Experimental Animals

The experiments with *Macaca mulatta* monkeys were performed at the Research Institute of Medical Primatology, National Research Center “Kurchatov’ Institute”, Sochi, Russia. The biochemical part of the study was carried out in the Department of Free Radical Biochemistry, E.I. Chazov’s National Medical Research Center of Cardiology, Russian Ministry of Health, Moscow, Russia.

The study used mature rhesus macaque (*Macaca mulatta*) males aging 4–5 years and weighing 4.5 ± 0.5 kg. The control and three experimental groups comprised n = 3 animals each. During the experiments, the monkeys were kept at ambient temperature of 24 ± 2 °C, relative humidity of 65 ± 5% and natural daylight in individual ventilated cages with automatic drinking bowls. The animals were adapted to environmental conditions and to the service personnel and maintained on pellets, which were balanced by all indices and supplemented with green fodder and fruits. The experiments on animals were performed in compliance with state standard N33218-2014, “Guidelines for the maintenance and care of laboratory animals. Rules for the maintenance and care of non-human primates”.

The blood specimens (15 mL) were taken in the morning after overnight fasting from the ulnar vein into the blood collection tubes containing EDTA (1 mg/mL) as an anticoagulant and antioxidant agent. The blood cells were separated by a refrigerated centrifuge Eppendorf 5702R (Eppendorf, Hamburg, Germany) at 1000× *g* for 10 min; thereupon the blood plasma was collected, frozen, and transported on dry ice for preparative isolation of LDL in the Department for Free Radical Biochemistry (Moscow, Russia).

### 4.2. LDL Preparative Isolation

Isolation of LDL was performed as described previously [31] using differential ultracentrifugation in an Optima XPN-80 Beckman ultracentrifuge equipped with a type 70.1 Ti fixed-angle rotor. The isolated LDL were dialyzed at 4 °C for 18 h against 2000 volumes of PBS (10 mM) at pH 7.4. Protein concentration in LDL preparation was estimated by the Lowry method [32]. The content of B-100 apoprotein was estimated on an Architect C8000 chemical analyzer (Abbot, Lake Forest, IL, USA) with the use of reagents from the same producer.

### 4.3. Preparation of FITC-Labeled LDL

Isolated LDL were labeled with fluorescein isothiocyanate (FITC) as described elsewhere [23,33]. To this end, the solid salt of sodium tetraborate was added to LDL preparations initially kept in isotonic phosphate buffer at pH 7.2 to elevate pH up to 9.2, and thereafter the solution was centrifuged for 10 min at 13,000× *g*. The LDL protein was determined in the supernatant. The acquired LDL preparations were supplemented with FITC dissolved in dimethyl sulfoxide to final concentration of B-100 apoprotein of 10 µg/1 mg; thereupon, the solution was rapidly mixed and kept in the dark for 10 min under periodic stirring. LDL clearance of their aggregates and excessive FITC was performed in a *gel filtration* column (18 × 1 cm) packed with Sephadex G-75. The FITC-labeled fractions were detected by the fluorescent spectra (excitation 495 nm, emission 519 nm) employing a Hitachi F-2700 (Japan).

### 4.4. Preparation of FITC-Labeled Dicarbonyl-Modified LDL

To harvest FITC-labeled dicarbonyl-modified LDL, the freshly prepared MDA solution was employed, which was obtained by the method of acid hydrolysis of 1,1,3,3-tetraethoxypropane (Sigma) [8,34]. MDA concentration was determined by optical density at 267 nm in a UV-2600 Shimadzu spectrophotometer (Japan) using a molar extinction coefficient of 31,800 M^−1^cm^−1^ [8,34]. Modification of FITC-labeled LDL with dicarbonyls was performed by incubation of LDL preparations for 3 h in the dark at 37 °C in the presence of freshly obtained MDA (*vide supra*), glyoxal, or methylglyoxal (all reagents from Sigma, St. Louis, MO, USA) using 1 micromol dicarbonyl per 100 µg LDL protein [8,11,23]. The effectiveness of modification of amino acid residues of proteins (including apoprotein 100) at high concentrations of dicarbonyls (such as MDA and methylglyoxal) is at least 80–90%, according to literature data [34,35]. The initial fluorescence of all samples injected into animals was identical because a single pool of FITC-labeled LDL was used to modify the LDL. Clearance of excessive dialdehydes was performed by dialysis at 4 °C for 18 h against 2000 volumes of 10 mM PBS at pH 7.4 [11,23].

### 4.5. Examination of Clearance of Dicarbonyl-Modified LDL in Macaca Mulatta Monkeys

The acquired specimens of FITC-labeled dicarbonyl-modified LDL were transported on ice to the *Research Institute of Medical Primatology* (*Sochi*) in a thermal container at 0 °C. Prior to administration, the FITC-labeled dicarbonyl-modified monkey-derived LDL solutions were sterilized by forcing them through the syringe filters with a pore diameter of 0.45 µm (Maine Manufacturing, Sanford, ME, USA) and then injected into tranquilized animals into the ulnar vein (200 µg LDL protein/kg body weight). At certain intervals (6 min, 1.5 h, 6 h, 24 h), the specimens of whole blood (1 mL) were taken and centrifuged in a refrigerated centrifuge Eppendorf 5702R (Austria) at 1000× *g* for 10 min; thereafter, the blood plasma was collected. The acquired plasma specimens were transported to the *Department for Free Radical Biochemistry* (*Moscow*) in a thermal container on ice at 0 °C for fluorescent analysis using an Hitachi F-2700 (Japan) spectrophotometer at excitation and emission wavelengths of 495 and 519 nm, respectively [23]. The fluorescence of the standard solution of dehydrated quinine sulfate (0.01 μg/mL in 0.05 M H_2_SO_4_) was taken as the unit of fluorescence intensity.

### 4.6. Clinical Study of the Effects of PCSK9 Inhibitor Evolocumab

The clinical study of the action of PCSK9 inhibitor evolocumab [24] enrolled men aging 59 ± 10 year (*n* = 9), who had stable coronary heart disease (CHD) and atherosclerotic lesion in at least one major artery documented by coronary angiography. The patients received standard therapy, which included antiaggregants, β-blockers, angiotensin-converting enzyme inhibitors, and antagonists of angiotensin receptors. Prior to examination, all the patients received the maximally tolerated dose of statins. Since the statin therapy did not attain the target levels of LDL cholesterol, the patients were treated with hypolipidemic therapy implicating a PCSK9 inhibitor, evolocumab (Amgen, Thousand Oaks, CA, USA), in a once-monthly dose of 420 mg. The indices of lipid metabolism were determined with routine methods employing an Architect C8000 Abbott (Abbott Park, IL, USA) chemical analyzer and the reagents of the same firm [24]. The plasma level of oxidatively modified LDL was established with an immunochemical method using oxidized LDL ELISA test kits (Mercodia, Uppsala, Sweden), containing monoclonal antibodies mAb-4E6 against MDA-modified LDL [25].

### 4.7. Statistics

The data were analyzed statistically using Statistica 10.0 software. Due to small data groups, significance was assessed with the non-parametric Mann–Whitney test at *p* < 0.05 for two independent samples. The clinical data were analyzed with MedCalc 5.8 software (MedCalc Software Ltd., Ostend, Belgium).

## 5. Conclusions

According to available data, dicarbonyl-modified LDL plays an important role in the molecular mechanisms of atherosclerotic lesions in the vascular wall and in endothelial dysfunction. In this connection, it seemed important to examine the clearance of MDA-modified LDL as well as glyoxal- and methylglyoxal-modified LDL in primates such as *Macaca mulatta* monkeys. The study showed that glyoxal- and methylglyoxal-modified LDL were eliminated from the blood flow of monkeys at the same rate as non-modified (native) LDL. In contrast, the content of MDA-modified LDL in monkey circulation dropped extremely rapidly as early as the first minutes post-injection. Administration of the PCSK9 inhibitor evolocumab to CHD patients, which up-regulates utilization of LDL in the liver, not only decreased the level of LDL cholesterol but also produced a comparative drop in the content of MDA-modified LDL. The present data can be used as a basis for novel approaches in the development of medicamentous therapies for atherosclerosis and diabetes mellitus.

## Figures and Tables

**Table 1:** Clearance of various dicarbonyl-modified LDL in the blood stream of *Macaca mulatta* monkeys at early follow-up. (n = 3; * differences from native LDL—*p* < 0.05). Results are presented in relative units of fluorescence per 1 mL of blood plasma.

Groups	Time	
	6 min	90 min
1. Natural LDL	10.33 ± 1.34	10.07 ± 1.50
2. Glyoxal-modified LDL	11.62 ± 0.53	11.36 ± 1.45
3. Methylglyoxal-modified LDL	12.42 ± 4.15	9.36 ± 1.64
4. MDA-modified LDL	1.27 ± 0.20 *	1.04 ± 0.16 *
